# Diverse Gene Cassette Arrays Prevail in Commensal *Escherichia coli* From Intensive Farming Swine in Four Provinces of China

**DOI:** 10.3389/fmicb.2020.565349

**Published:** 2020-10-14

**Authors:** Xiuping Zhang, Xinxin Li, Weihua Wang, Jiali Qi, Dong Wang, Lei Xu, Yong Liu, Yanming Zhang, Kangkang Guo

**Affiliations:** ^1^College of Veterinary Medicine, Northwest A&F University, Yangling, China; ^2^College of Animal Science, Tarim University, Alar, China; ^3^Weinan Vocational and Technical College, Weinan, China; ^4^College of Life Science, Northwest A&F University, Yangling, China

**Keywords:** antimicrobial resistance, multiple-drug resistance, class 1 integrons, gene cassette, commensal *Escherichia coli*, antibiotic-resistant bacteria, antibiotic-resistant genes

## Abstract

Multiple-drug resistance bacteria containing antimicrobial resistance genes (ARGs) are a concern for public health. Integrons are bacterial genetic elements that can capture, rearrange, and express mobile gene cassettes responsible for the spread of ARGs. Few studies link genotype and phenotype of swine-related ARGs in the context of mobile gene cassette arrays among commensal *Escherichia coli* (*E. coli*) in nonclinical livestock isolates from intensive farms. In the present study, a total of 264 isolates were obtained from 330 rectal swabs to determine the prevalence and characteristics of antibiotic-resistant gene being carried by commensal *E. coli* in the healthy swine from four intensive farms at Anhui, Hebei, Shanxi, and Shaanxi, in China. Antimicrobial resistance phenotypes of the recovered isolates were determined for 19 antimicrobials. The *E. coli* isolates were commonly nonsusceptible to doxycycline (75.8%), tetracycline (73.5%), sulfamethoxazole-trimethoprim (71.6%), amoxicillin (68.2%), sulfasalazine (67.1%), ampicillin (58.0%), florfenicol (56.1%), and streptomycin (53.0%), but all isolates were susceptible to imipenem (100%). Isolates [184 (69.7%)] exhibited multiple drug resistance with 11 patterns. Moreover, 197 isolates (74.6%) were detected carrying the integron-integrase gene (*intI*1) of class 1 integrons. A higher incidence of antimicrobial resistance was observed in the *intI*1-positive *E. coli* isolates than in the *intI*1-negative *E. coli* isolates. Furthermore, there were 17 kinds of gene cassette arrays in the 70 integrons as detected by sequencing amplicons of variable regions, with 66 isolates (94.3%) expressing their gene cassettes encoding for multiple drug resistance phenotypes for streptomycin, neomycin, gentamicin, kanamycin, amikacin, sulfamethoxazole-trimethoprim, sulfasalazine, and florfenicol. Notably, due to harboring multiple, hybrid, and recombination cassettes, complex cassette arrays were attributed to multiple drug resistance patterns than simple arrays. In conclusion, we demonstrated that the prevalence of multiple drug resistance and the incidence of class 1 integrons were 69.7 and 74.6% in commensal *E. coli* isolated from healthy swine, which were lower in frequency than that previously reported in China.

## Introduction

Antimicrobial-resistant bacteria, especially multiple drug-resistant (MDR) strains have caused many outbreaks of food-borne diseases and infectious diseases worldwide, threatening human and animal health (Paitan, 2018). Integrons located on either chromosome or mobile genetic elements (MGEs), such as plasmids and transposons, are considered responsible for the horizontal gene transfer of antimicrobial resistance (AMR; Stalder et al., 2012). Integrons are natural recombination and expression systems with the ability to acquire gene cassettes (Partridge et al., 2018). Gene cassettes are a major source of the resistance genes found in clinical, commensal, and environmental isolates of bacteria. A gene cassette is a small mobile element (0.5–1 kb) consisting of a single gene (occasionally two) and a recombination site (*attC*; Partridge et al., 2009). Gene cassettes encode proteins that facilitate interactions with their extracellular environment (Timothy et al., 2019). Several cassettes may be inserted into the same integron forming a tandem array. Class 1 integrons in bacteria ubiquitously reside in gastrointestinal tracts of animals and humans, and their abundance and genetic diversity can readily change in response to environmental pressures (Alonso et al., 2017). The class 1 integrons are diverse and significant players in the spread of AMR. *E. coli* has been shown to be a significant reservoir of genes encoding for AMR and has been suggested as a useful indicator for resistance in bacterial communities (Ge et al., 2020; Holcomb and Stewart, 2020; Zhang et al., 2020b). Highly dynamic and diverse of *E. coli* populations exist in the swine intestinal microbiota and in the farm environment throughout the full production cycle, suggesting the potential for carriage of antimicrobial resistance and the presence of clinical integrons (Marchant and Morenoa, 2013; Van den Meersche et al., 2020).

Development of MDR in intestinal flora is closely associated with integrons and their gene cassettes (Olivier et al., 2018). Integrons are genetic platforms for captured gene cassettes, which are regarded as adaptation and evolution of bacteria (José, 2018). They have traditional genetic structure of two conserved segments (5'-CS and 3'-CS) and a variable region (Barraud and Ploy, 2015). At least 130 different (<98% identical) cassettes that carry known or predicted antibiotic resistance genes have been identified, along with many cassettes of unknown function (Partridge et al., 2009). Class 1 integrons play a crucial role in the propagation of ARGs, and have been surveyed in numerous ecosystems and animals. Zhang et al. found the average content of class I integrons as 1.31 × 10^4^ copies/100 ml in drinking water from 71 cities in China (Zhang et al., 2020a). However, the data are still lacking on the epidemiology of class 1 integrons and integron-borne gene cassettes in commensal *E. coli* among livestock herds, in particular at the source of pork production.

To promote effective antimicrobial stewardship, the government of China issued the National Action Plan to Contain Animal Original Antimicrobial Resistance (2017–2020) on June 22, 2017. This action is in line with the strategic objectives of WHO’s Global Action Plan on antimicrobial resistance. Tracking and surveillance of antimicrobial resistant bacteria in livestock is a critical step toward protecting humans and animals from infections. Anhui, Hebei, Shanxi, and Shaanxi provinces are mainly livestock-raising areas in China, with the amount of fattening swine for market (unit: million heads) of 283.74, 370.96, 81.46, 115.08, with the number of stocks at year-end (unit: million heads) of 135.63, 182.08, 54.95, 83.9, respectively, in 2019 (NBSC, 2019). However, data of antimicrobial resistance in livestock are inadequate in these areas.

The aim of the present study was to obtain the phenotypic and genotypic characterization of selected antimicrobial resistance determinants found in commensal *E. coli* isolated from intensively farmed swine. We detected the MDR phenotypic profiles, the abundance of class 1 integrons, and gene cassettes of class 1 integrons through antimicrobial susceptibility testing, PCR assay, and DNA sequencing.

## Materials and Methods

### Sampling and Isolation

From March to May, 2019, a total of 330 rectal sterile swabs were collected from healthy growing-finishing swine of four intensive farms in Anhui, Hebei, Shanxi, and Shaanxi province, China (Table 1). The annual production of the intensive farms was 1,000–1,200 heads (i.e., the breeding stock was 500–600 heads), with growing-finishing swine (mixed sex, same aged from 70 to 185 days) raised in the mode of all-in and all-out. Random sampling was conducted on 90‐ to 180-days growing-finishing swine in 10% of the breeding stock herds, with one swab per swine. Healthy swines were determined by observation indicators of their good physical and mental state, with normal body temperature, appearance, and behavior, feed intake and drinking, and excretion. The Animal Welfare and Research Ethics Committee of Northwest A&F University (Yangling, China) approved the protocol of the experiment (protocol number: NWAFUSM2018005). All the rectal sterile swabs were placed into an ice box and transferred to the laboratory within 6 h for further bacteriological analysis.

**Table 1:** Samples from Anhui, Hebei, Shanxi, and Shaanxi.

Time^a^	Anhui	Hebei	Shanxi	Shaanxi
March	30	30	30	30
April	30	30	30	30
May	20^b^	20^b^	20^b^	30
Total	80	80	80	90

a Sampling was originally planned on the 1st, 15th, 30th of March, April, and May in each farm, when the ages of the growing-finishing swine varied from 90 to 180 days. In total, 30 samples were to be collected in each farm every month.

b Sampling plan was not conducted on the 30th of May in Anhui, Hebei, and Shanxi, so only 20 samples were obtained from their farms, respectively.

Isolation and identification of *E. coli* were performed with rectal sterile swabs and transferred to sterile culture tubes containing 5 ml of Luria-Bertani (LB) broth and mixed vigorously (200 r/min) at 37°C for 8 h. After enrichment, a loop of LB broth culture was streaked onto eosin-methylene blue medium (EMB) agar and incubated at 37°C for 24 h. Colonies showing a metallic sheen were considered presumptive *E. coli* isolates, and positive colonies were chosen for further biochemical identification using Gram-negative identification cards of an automated VITEK2 microbial identification system (BioMerieux, France), according to the manufacturers.

### Antimicrobial Susceptibility Testing

All *E. coli* isolates were tested susceptible to seven classes of antimicrobials by the Kirby-Bauer disk diffusion method on Mueller-Hinton (MH) agar plates following Clinical and Laboratory Standards Institute (CLSI) procedures (CLSI, 2017). A panel of 19 antimicrobial agents (Table 2) are commonly used against clinical infections. In animal husbandry, discs containing streptomycin (STR, 10 μg), neomycin (NEO, 30 μg), gentamicin (GEN, 10 μg), kanamycin (K, 30 μg), amikacin (AK, 30 μg), amoxicillin (AMC, 20 μg), ampicillin (AMP, 10 μg), cephalexin (CL, 30 μg), cefotaxime (CTX, 30 μg), imipenem (IMP, 10 μg), ciprofloxacin (CIP, 5 μg), enrofloxacin (ENR, 10 μg), norfloxacin (NOR, 10 μg), tetracycline (TET, 30 μg), doxycycline (DX, 30 μg), sulfamethoxazole-trimethoprim (SMZ/TMP, 25 μg), sulfasalazine (SIZ, 300 μg), erythromycin (ERY, 15 μg), and florfenicol (FF, 30 μg) were used. The reference strain *E. coli* ATCC 25922 was used for quality control. Susceptibility decision was described as resistant (R), intermediate (I), or susceptible (S) as the CLSI and the sensitivity criteria for *Enterobacteriaceae* (Hangzhou Microbiology Co., Ltd., China, 2019). Isolates were considered as nonsusceptible when reported as either intermediate susceptible (I) or resistant (R; Jarlier et al., 2019). *E. coli* isolates exhibiting MDR was defined as acquired nonsusceptible to ≥1 agent in ≥3 antimicrobial categories (Magiorakos et al., 2012).

**Table 2:** Antimicrobial agents were used to define the susceptibility of *Escherichia coli* isolates.

Antimicrobials*^a^*		Content (μg/disc)	Breakpoint (mm)		
			R*^b^*	I*^b^*	S*^b^*
Aminoglycosides	Streptomycin (STR)	10	≤12	13–14	≥15
	Neomycin (NEO)	30	≤12	13–16	≥17
	Gentamicin (GEN)	10	≤13	14–17	≥18
	Kanamycin (K)	30	≤13	14–17	≥18
	Amikacin (AK)	30	≤14	15–16	≥17
β-Lactams	Amoxicillin (AMC)	20	≤13	14–17	≥18
	Ampicillin (AMP)	10	≤13	14–16	≥17
	Cephalexin (CL)	30	≤14	15–17	≥18
	Cefotaxime (CTX)	30	≤14	15–22	≥23
	Imipenem (IPM)	10	≤13	14 15	≥16
Quinolones	Ciprofloxacin (CIP)	5	≤15	16–20	≥21
	Enrofloxacin (ENR)	10	≤21	22–28	≥29
	Norfloxacin (NOR)	10	≤12	13–16	≥17
Tetracyclines	Tetracycline (TET)	30	≤14	15–18	≥19
	Doxycycline (DX)	30	≤12	13–15	≥16
Sulfonamides	Sulfamethaxazole/trimethoprim (SMZ/TMP)	25	≤10	11–15	≥16
	Sulfasalazine (SIZ)	300	≤12	13–16	≥17
Macrolides	Erythromycin (ERY)	15	≤13	14–22	≥23
Chloramphenicol	Florfenicol (FF)	30	≤12	13–17	≥18

a Veterinary antibiotics were chosen for testing including aminoglycoside (streptomycin STR, neomycin NEO, gentamicin GEN, kanamycin K, amikacin AK), β-lactam (amoxicillin AMC and ampicillin AMP, cephalexin CL, cefotaxime CTX, mipenem IPM), quinolone (ciprofloxacin CIP, for enrofloxacin ENR and norfloxacin NOR), tetracycline (tetracycline TET, doxycycline DX), sulfonamide (sulfamethoxazole-trimethoprim SMZ/TMP, and sulfasalazine SIZ), macrolides (erythromycin ERY), and chloramphenicol (florfenicol FF).

b R stands for resistant, I for intermediate resistant, S for susceptible.

### PCR Detection of *intl*1 Gene in *E. coli* Isolates

All *E. coli* isolates were screened by PCR for the class 1 integron-integrase (*intl*1) gene to confirm the presence of class 1 integrons. The primers were *intI*1 F 5'-ACGAGCGCAAGGTTTCGGT-3' and *intI1* R 5'-GAAAGGTCTGGTCATACATG-3' (Bass et al., 1999). The amplification protocol was performed as follows: initial denaturation (94°C for 5 min), followed by 30 cycles of denaturation (94°C for 30 s), annealing (56°C for 30 s), extension (72°C for 2 min), then a final extension (72°C for 10 min). Amplicons were separated by electrophoresis in a 1.5% agarose gel and sequenced (Beijing Qingke Biotech Co., Ltd. China).

### Arrangement of Resistance Gene Cassettes in Class 1 Integrons

The variable regions of the integron-positive isolates were further amplified for gene cassettes by PCR using primers of *intI1-*V FP 5'-TCATGGCTTGTTATGACTGT-3' and *intI-*V RP 5'-GTAGGGCTTATTATGCACGC-3' (White et al., 2000). PCR conditions were the same as above except for the annealing temperatures for the fragments. After separated by electrophoresis in 1.5% agarose gel, the gel-recovered product was ligated with pMD19-T vector (TaKaRa Bio Group, Japan) at 16°C for 4 h and then transferred into *E. coli* DH5α cells (QiaGen Biotech CO., LTD, China). The *E. coli* DH5α cultures were inoculated on the LB agar plates supplemented with 100 μg/ml ampicillin. The amplified fragments of different sizes were sequenced. The nucleotide sequences were analyzed by using Nucleotide BLAST on NCBI website^1^ and the integron database INTEGRALL.^2^ Standard Nucleotide BLAST was set as Search database Nucleotide collection (nr/nt) using Megablast (optimized for highly similar sequences). The general parameters were set by selecting the maximum number of aligned sequences to display (100) and expected threshold (10). Once the nucleotide BLAST results were available, pairwise and CDS features were chosen for alignment view.

### Statistical Analysis

Data were analyzed using the Statistical Product and Service Solutions software (SPSS, version 20.0). Chi square test and Fisher’s exact test of analysis of variance (ANOVA) were used to determine the statistical significance of data. Values were considered as having a statistically significant difference if *p* < 0.05 and as an extremely distinct difference if *p* < 0.01.

## Result

### Antimicrobial Resistance Profiles of *E. coli* in Four Provinces

**Figure 1:**
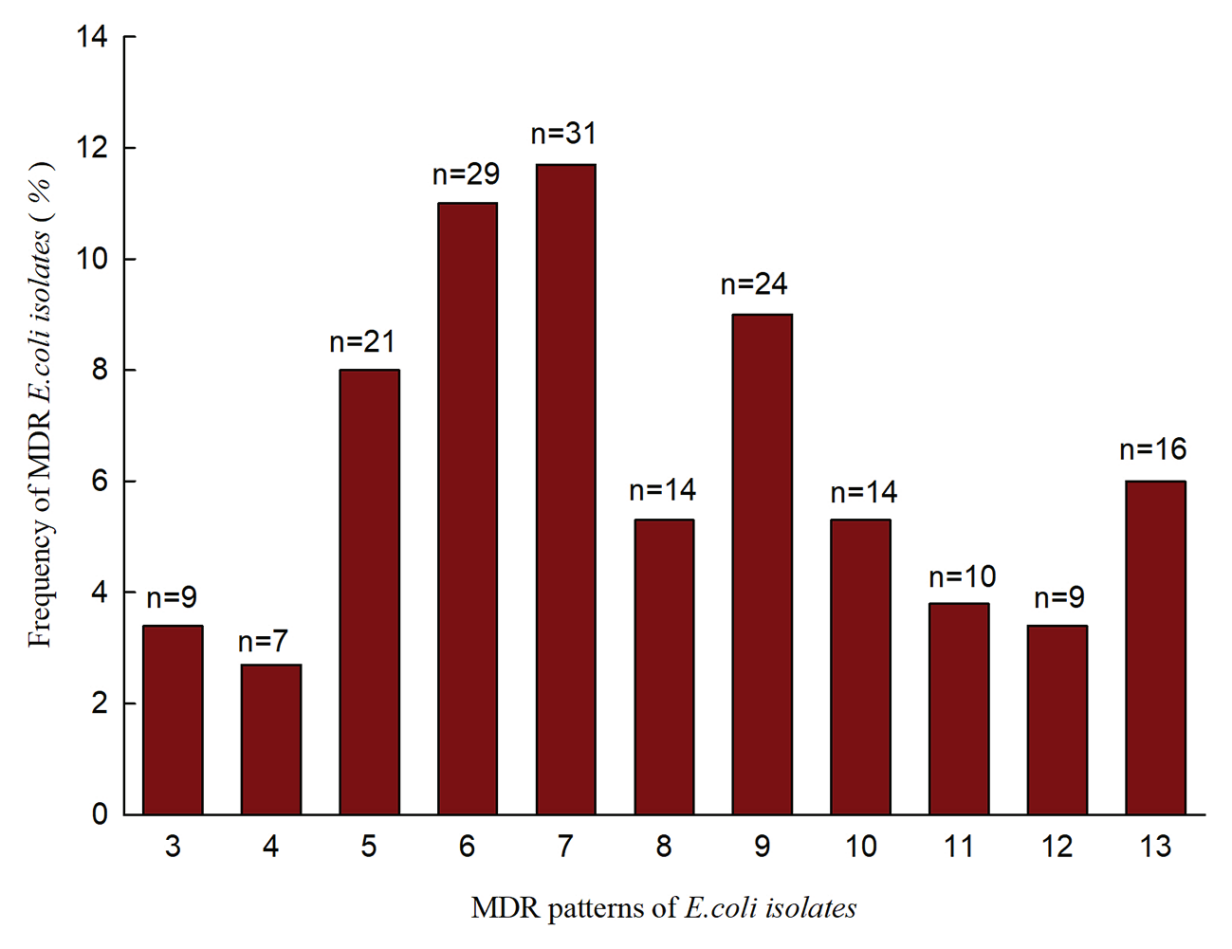
Antimicrobial resistance pattern and frequency of 184 MDR *E. coli* isolates.

In our study, we investigated the prevalence of antimicrobial resistance in 264 *E. coli* isolates recovered from rectal swabs. They were resistant to at least one antimicrobial agent (Table 3, left). A high proportion of *E. coli* isolates were nonsusceptible to doxycycline (75.8%), tetracycline (73.5%), sulfamethoxazole-trimethoprim (71.6%), amoxicillin (68.2%), sulfasalazine (67.1%), ampicillin (58.0%), florfenicol (56.1%), and streptomycin (53.0%). However, all isolates were susceptible to imipenem. A total of 184 (69.7%) *E. coli* isolates exhibited MDR, with 11 MDR patterns (Figure 1). Importantly, 16 *E. coli* isolates (6.0%) displayed resistance against 13 antimicrobial agents. The prevalence of *E. coli* MDR in Anhui, Hebei, Shanxi, and Shaanxi were 78.6, 80, 74.4, and 58.0%, respectively (Table 4).

**Table 3:** Frequency of antimicrobial susceptibility and *intI1* gene in 264 *E. coli* isolates.

Antimicrobials	Numbers of *E. coli* isolates (%)				
	Nonsusceptible	Susceptible	*intI1*+*^a^* (*n* = 197)	*intI1*–*^a^* (*n* = 67)	*P**^b^*
Doxycycline	200 (75.8)	64 (24.2)	138 (70.1)	28 (14.2)	0.000
Tetracycline	194 (73.5)	70 (26.5)	136 (69.0)	32 (16.2)	0.000
Sulfamethaxazole/trimethoprim	189 (71.6)	75 (28.4)	132 (67.0)	20 (10.2)	0.005
Amoxicillin	180 (68.2)	84 (31.8)	131 (66.5)	14 (7.1)	0.000
Sulfasalazine	177 (67.1)	87 (32.9)	127 (64.5)	30 (15.2)	0.313
Ampicillin	153 (58.0)	111 (42.0)	105 (53.3)	7 (3.6)	0.000
Florfenicol	148 (56.1)	116 (43.9)	107 (54.3)	21 (10.7)	0.000
Streptomycin	140 (53.0)	124 (47.0)	75 (38.1)	10 (5.1)	0.000
Erythromycin	128 (48.5)	136 (51.5)	47 (23.9)	12 (6.1)	0.001
Enrofloxacin	113 (46.6)	141 (53.4)	74 (37.6)	12 (6.1)	0.003
Neomycin	89 (33.7)	175 (66.3)	55 (27.9)	4 (2.0)	0.000
Kanamycin	87 (33.0)	177 (67.0)	59 (29.9)	12 (6.1)	0.055
Cephalexin	86 (32.6)	178 (67.4)	50 (25.4)	0 (0.0)	0.000
Ciprofloxacin	63 (23.9)	201 (76.1)	34 (17.3)	1 (0.5)	0.001
Gentamicin	57 (21.6)	207 (78.4)	25 (12.7)	0 (0.0)	0.002
Norfloxacin	53 (21.1)	211 (79.9)	27 (13.7)	3 (1.5)	0.040
Amikacin	46 (17.4)	218 (82.6)	21 (10.7)	3 (1.5)	0.128
Cefotaxime	44 (16.7)	220 (83.3)	24 (12.2)	0 (0.0)	0.003
Imipenem	0 (0)	264 (100.0)	-*^d^*	-	-
Multiple Drugs*^c^*			155 (78.7)	29 (43.3)	0.000

a *intI1*+ stands for *intI1* gene-positive isolate and *intI1*− for *intI1* gene-negative isolate.

b *p* means the difference between *intI1*-positive isolate and intI1-negative isolate. Chi square test and Fisher’s exact test were used.

c Multiple drugs meant at least more than three antimicrobial agents.

d intI1 was not detected in *E. coli* isolates susceptible to imipenem.

**Table 4:** Distribution of multiple drug-resistant (MDR), class 1 integrons, and gene cassettes among *E. coli* isolates in Anhui, Hebei, Shanxi, and Shaanxi.

Characteristic	Isolate No. (%)				
	Anhui (*n* = 56)	Hebei (*n* = 30)	Shanxi (*n* = 78)	Shaanxi (*n* = 100)	Total (*n* = 264)
MDR	44 (78.6)	24 (80.0)	58 (74.4)	58 (58.0)	184 (69.7)
*Intl*1	50 (89.2)	25 (83.3)	58 (74.4)	64 (64.0)	197 (74.6)
MDR-*intl*1	40 (71.4)	19 (63.3)	52 (66.7)	44 (44.0)	155 (58.7)
*intl*1-gene cassette-MDR	19 (33.9)	11 (36.7)	10 (12.8)	30 (30.0)	70 (26.5)

### The Incidence of Class 1 Integrons and Their Association With Antimicrobial Resistance in *E. coli* Isolates

The *IntI*1 gene was detected in 197 (74.6%) *E. coli* isolates, including 155 (78.7%) MDR isolates. I*ntI*1-positive *E. coli* isolates were most commonly nonsusceptible to doxycycline (70.1%), tetracycline (69.0%), sulfamethoxazole-trimethoprim (67.0%), amoxicillin (66.5%), sulfasalazine (64.5%), florfenicol (54.3%), and ampicillin (53.3%). There were significant differences in AMR between *intI*1-positive *E. coli* isolates and *intI*1-negative *E. coli* isolates, not only in single antimicrobial resistance but also in multiple drugs resistance (*p* < 0.01), except sulfasalazine (*p* = 0.313), kanamycin (*p* = 0.055), and amikacin (*p* = 0.128).

After sequencing the variable regions of class 1 integrons, we found 17 gene cassette arrays and 10 gene cassettes (*aadA1*, *aadA2*, *aadA5*, *aadA16*; *dfrA1*, *dfrA7*, *dfrA12*, *dfrA17*, *cmlA1*, and *orfF*) in 70 *E. coli* isolates (Table 5). Among them, 66 (85.7%) isolates had gene cassettes that corresponded with respective phenotypic MDR patterns, encoding resistance to streptomycin, neomycin, gentamicin, kanamycin, amikacin, sulfamethoxazole-trimethoprim, sulfasalazine, and florfenicol. However, there were three *E. coli* isolates that did not express their cassette arrays for MDR patterns at all, including *dfrA12-orfF-aadA2/1*, *aadA1-aadA2*, *aadA2*, and *aadA1* (Table 5, marked with lower case c). Different gene cassette arrays, even from the same array showed different phenotypic patterns of MDR. In addition, prevailing arrays were different in the four farms: five arrays in Anhui (*n* = 19), five arrays in Hebei (*n* = 11), eight arrays in Shanxi (*n* = 10), and six arrays in Shaanxi (*n* = 30).

**Table 5:** Characterization of cassette arrays and integron-associated antibiotic resistance in 70 isolates of *E. coli*.

Gene cassette array		Resistance phenotype*^a^*	No. of antimicrobials	Amplicon size (bp)	Isolate number	sites	Frequency (*n* = 197,%)
1	*dfrA17–aadA5*	TET + AMC + ***NEO*** + AMP + DX + ***SMZ/TMP***	6	1,778	5	Anhui	19 (9.6)
		ERY + TET + AMC + ***NEO*** + AMP + DX + ***SMZ/TMP*** ^b^	7	1,780	11	Shaanxi	
		TET + ***NEO*** + ENR + DX + ***STR*** + NOR + ***SMZ/TMP***	7	1,787	1	Shanxi	
		ERY + TET + AMC + ***NEO*** + ***GEN*** + DX + ***SMZ/TMP***	7	1,794	1		
		ERY + TET + ***STR***	3	1,794	1		
2	*dfrA12–orfF–aadA2*	ERY + TET + AMC + FF + CL + ***STR***	6	2,035	1		12 (6.0)
		ERY + TET + AMC + ENR + ***GEN*** + AMP + DX + ***SMZ/TMP***	8	2,035	1	Shaanxi	
		ERY + TET + AMC + ENR + DX + AMP + ***SMZ/TMP***	7	2,036	4		
		ERY + TET + AMC + ***NEO*** + AMP + DX + ***SMZ/TMP*** ^b^	7	2,037	5		
		STR + ***NEO*** + ***AK*** + AMC + AMP + CL + CIP + ENR + TET + DX + ***SIZ*** + ***SMZ/TMP***	12	2,070	1	Hebei	
3	*dfrA12–orfF–aadA2–adA1–cmlA1*	***STR*** + ***NEO*** + ***AK*** + AMC + AMP + CL + CIP + ENR + TET + ***SMZ/TMP*** + ***SIZ*** + ERY + ***FF***	13	2,084	8	Anhui	8 (4.0)
4	*dfrA12–orfF–aadA2/aadA2–aadA1–cmlA1**^c^*	TET + AMC + **FF** + ***GEN*** + AMP + DX + ***SMZ/TMP***	7	2,054	2	Hebei	2 (1.0)
5	*dfrA12–orfF–aadA2/aadA1–cmlA1*^c^	ERY + TET + AMC + AMP + DX + ***STR*** *+* ***SMZ/TMP***	7	2,036	1	Shaanxi	2 (1.0)
		TET + AMC + AMP + DX^d^	4	2,026^e^	1	Shanxi	
6	*dfrA12–orfF–aadA2/1**^c^*	ERY + TET + AMC + ***FF*** + **GEN** + AMP + CL + DX + ***STR*** + CTX + ***SMZ/TMP***	11	2,026	1		1 (0.5)
7	*dfrA12–orf F*	CL + ***K*** + ***SMZ/TMP***	3	1,197	3	Hebei	3 (1.5)
8	*dfrA7–aadA1-2–aadA1-1**^c^*	ERY + TET + AMC + ***NEO*** + ***GEN*** + AMP + ENR + DX + ***STR*** + ***SMZ/TMP***	10	1,175	2	Shaanxi	2 (1.0)
9	*dfrA1–aadA2/1*^c^	TET + AMC + FF + DX + ***SMZ/TMP***	5	1,735	4		5 (2.5)
		***STR*** + ***AK*** + AMC + AMP + NOR + CL + ENR + TET + DX + SIZ + ERY + FF + ***SMZ/TMP***	13	1,699	1	Shanxi	
10	*dfrA1–aadA2*	ERY + TET + ***NEO*** + DX + ***STR***	5	1,685	4	Hebei	4 (2.0)
11	*dfrA1–aadA1*	TET + AMC + ***NEO*** + FF + ***SMZ/TMP***	5	1,706	1	Anhui	1 (0.5)
12	*aadA1–dfrA1–aadA2*	ERY + TET + AMC + ***GEN*** + AMP + DX + ***SMZ/TMP***	7	1,709	4		4 (2.0)
13	*aadA1–dfrA1*	ERY + TET + AMC + ***NEO*** + DX + NOR + ***STR*** + ***SMZ/TMP***	8	1,699	2	Shanxi	2 (1.0)
14	*aadA2*	ERY + TET + AMC + ***NEO*** + ***FF*** + ***GEN*** + AMP + ***K*** + ***STR*** + ***SMZ/TMP***	10	1,174	2	Shaanxi	2 (1.0)
		TET + AMC + AMP + DX^d^	4	1,122^e^	-	Shanxi	
15	*aadA16–aadA5–dfrA17*	ERY + TET + AMC + ENR + AMP + DX + ***SMZ/TMP*** + NOR	8	1,181	1		1 (0.5)
16	*aadA1–aadA2*	CL + CTX^d^	2	1,155	1	Hebei	1 (0.5)
17	*aadA1*	ERY + TET^d^	2	1,124	1	Anhui	1 (0.5)

a Italic bold means phenotypic resistance could track their gene cassettes.

b Two different gene cassette arrays have the same phenotypic MDR patterns.

c aadA2/1 or A2/aadA1 hybrid harbored in these arrays.

d Gene array was not exhibited in their corresponding phenotype(s) of antimicrobial agents.

e Two amplicons were in the same isolate.

Cassette arrays showed the following characteristics. First, the top three cassette arrays were *dfrA17–aadA5* (9.6%), *dfrA12–orfF–aadA2* (6.0%), and *dfrA12–orfF-aadA2–adA1–cmlA1* (4.0%). They were confirmed by the same or similar sizes of amplicons, but they exhibited different patterns of phenotypical MDR. This phenomenon also occurred in *dfrA1–aadA2/1* (2.5%), *aadA2* (1.5%), and *dfrA12–orfF–aadA2/1* (1.0%). Second, in six arrays, *E. coli* isolates with *aadA2/1* and *aadA2/aadA2* hybrid gene cassettes exhibited more complex MDR, such as *dfrA1–aadA2/1*, *dfrA7–aadA1-2–aadA1-1, dfrA12–orfF–aadA2/aadA2–aadA1–cmlA1*, *dfrA12-orfF–aadA2/aadA1–cmlA1*, *dfrA12–orfF–aadA2/aadA1–cmlA1*, and *dfrA12–orfF–aadA2/1*. Third, *E. coli* isolates with simple cassette arrays performed poorly on MDR, including *dfrA12–orfF*, *dfrA1–aadA2*, *dfrA1–aadA1*, *aadA1–dfrA1*, *aadA1–aadA2*, *aadA2*, and *aadA1*. In addition, one isolate yielded two amplicons, 2,026 and 1,122 bp, and they formed into two arrays of *dfrA12–orfF–aadA2/aadA1–cmlA1* and *aadA2*. Another special array, *aadA16–aadA5–dfrA17* was found for the first time.

## Discussion

### A Lower and Still Challenging Prevalence of Antimicrobial Resistance *E. coli* in Anhui, Hebei, Shanxi, and Shaanxi

In the present study, we reported the frequency of MDR and the incidence of class 1 integrons from nonclinical swine and were 69.7 and 74.6% in commensal *E. coli*, which was lower than those that had been reported among swine origin nonclinical *E. coli* in several provinces of China, such as Sichuan (99.23, 87.69%; Lin et al., 2014), Xiamen (100, 92.2%; Liu et al., 2015), Harbin (100, 91.67%; Zhang et al., 2013), Hulunbeir (77.8, 95.59%; Qiu, 2015), Liaoning (92.31, 71.43%; Zhao et al., 2017), and Yunnan (96.15, 84.62%; Li et al., 2019) between 2010 and 2019. This indicates that there is a clear difference in prevalence between the previous investigated districts and the four provinces. Antimicrobial application is responsible for emergence and spread of MDR intestinal bacteria in animal husbandry (Matjuda and Aiyegoro, 2016). Veterinary antimicrobials always have the most priority treatment, and some of them are often taken as growth promoters for swine fattening (Ochoa et al., 2016). Increasing diversity and abundance of the antimicrobial resistance in *E. coli* clinical isolates witnessed the extensive and excessive usage of veterinary antimicrobials in swine herds of 18 provinces in China between 2010 and 2019 (Zhang et al., 2017, 2019a; Yang et al., 2019). Swine farming could lead to enhanced concentration levels of various veterinary antimicrobials and ARGs in groundwater and soils (Qiao et al., 2018). The increasing prevalence of MDR *E. coli* and other antimicrobial-resistant microorganisms causes unpredictable consequences to the environment and humans. In China, alternative strategies have been effectively explored to combat veterinary antimicrobial resistance, such as vaccination, chicken egg yolk antibodies (Zhang et al., 2011), lactic acid bacteria (Yang et al., 2015), antimicrobial peptides (Kang et al., 2017), bacteriocins (Qian et al., 2020), antibiotic adjuvants (Liu et al., 2019), phytocompounds (Chang et al., 2019), and metal-based nanoparticles (Ali et al., 2020). In addition, the implementation of management strategies has been done to reduce antimicrobial usage in animal husbandry, including good hygiene practice and biosecurity measures (Hu and Cowling, 2020). Therefore, it is possible that the frequency of MDR and the incidence of class 1 integrons in the present intensive farms are in the decline process due to good veterinary hygiene managements and reduced antimicrobial usage.

Phenotypic susceptibility testing showed that doxycycline, tetracycline, sulfamethaxazole/trimethoprim, amoxicillin, sulfasalazine, florfenicol, and ampicillin resistance are still challenging intensive farms in Anhui, Hebei, Shanxi, and Shaanxi. Many studies pointed out that *E. coli* strains from swine farms had formed high resistance to the first‐ or the second-generation antibiotics, and some of the original drugs had lost their antibacterial effect, such as streptomycin, tetracycline, cephalothin, ampicillin, ofloxacin, and sulfamethoxazole (Tang, 2014; Zhang et al., 2017). The evolution of *E. coli* antimicrobial resistance is closely correlated with the emergence and dissemination of specific ARGs (Jiang et al., 2017a; Divya and Hatha, 2019; Wang et al., 2019) and virulence genes (VGs; Wang et al., 2010; Cheng et al., 2020). This indicates that commensal *E. coli* in healthy swine, with resistance against doxycycline, tetracycline, sulfamethaxazole/trimethoprim, amoxicillin, sulfasalazine, florfenicol, and ampicillin, may easily obtain antibiotic resistance genes and virulence genes that jeopardize swine health.

Notably, 11 isolates were highly resistant to florfenicol for carrying the *cmlA* gene (in arrays of *dfrA12–orfF–aadA2–adA1–cmlA1*, *dfrA12–orfF–aadA2/aadA2–aadA1–cmlA1*, and *dfrA12–orfF–aadA2/aadA1–cmlA1*), which encodes a specific chloramphenicol transporter. Florfenicol, a fluorinated chloramphenicol derivative, has been widely used against both Gram-positive and Gram-negative bacteria (Belaynehe et al., 2018). Another notable antimicrobial agent is imipenem, belonging to the carbapenems antibiotics, which has a broader range of activity against Gram-negative and Gram-positive bacteria. The carbapenem-resistant genes (*blaOXA-48*, *blaGES-1*, *blaKPC-2*, and *blaNDM-1*) were considered to be the most harmful to human health (Yang et al., 2016). In China, KPC-2, NDM, and OXA-48-like carbapenemases were predominant among the carbapenem-resistant *Enterobacteriaceae* (CRE) isolates from adult and children patients (Han et al., 2020). However, in the present study, all *E. coli* isolates were susceptible to imipenem, and carbapenem-resistant genes were not detected by PCR. It demonstrates that the carbapenem-resistant genes did not exist or was extremely low in the investigated farms, which is consistent with the carbapenem-resistant pollution that is not severe in the drinking water of China (Xin et al., 2019).

### Diverse Gene Cassette Arrays Are Common Among Intensive Farms

In the present study, the prevalence of MDR *E. coli* is closely associated with the presence of class 1 integrons. Our findings are in agreement with the previous studies that class 1 integrons are the most ubiquitous classes of integrons in the enteric bacteria (Stokes et al., 2001). According to the study of Yohann Lacotte, in the stress-free environmental settings of *E. coli*, class 1 integrons live a relaxed life at low-cost structure, which can favor their maintenance and prevalence in cassette networks (Lacotte et al., 2017). In the present study, the healthy swine gut meant a safe environment for the low-cost class 1 integrons, thus 74.6 % (*n* = 197) integrons were highly prevalent in common *E. coli* isolates. They can offer a wider platform for the acquisition, rearrangement, and expression of gene cassettes. In addition, isolates (*n* = 29) without integrons also showed MDR, suggesting that other determinants might contribute to their resistance. Farm animals and manure are a source of food-borne and water-borne human pathogens (Bailey et al., 2011). Being a DNA pollutant (Gillings, 2018), the abundance of *intI*1 ranged from 3.83 × 10^−4^ to 4.26 × 10^0^ *intI*1/cell in eight ecosystems, even in giant pandas (47%) and remote rural area animals (6.7%) in China (Ma et al., 2017; Rehman et al., 2017; Zou et al., 2018). Xia et al. reviewed that *E. coli* had the highest positive rates (65.4%) of integrons from human patients with Gram-negative bacteria isolates in China during 2000–2014 (Xia et al., 2016). It suggests that MDR *E. coli* with a high number of class 1 integrons were widely distributed in swine farms, also indicating a higher incidence of lateral gene-transfer events.

In the present study, 17 gene cassette arrays in the commensal *E. coli* isolates were more diverse than those that had been reported in different animals and their products during the past 10 years in China, Spain, and Australia. There are nine arrays that are summarized from swine farming settings and pork products (*dfrA1–aadA1*, *aadA22*, *dfr17–aadA5*, *dfrA12–orfF–aadA2*, *dfrXII–orfF–aadA2*, *aadA2*, *dfrA1–catB3–aacA4*, *aadB–aadA2*, and *dfrA12–aadA2–cmlA1–aadA1*; Chen, 2013; Wei, 2014; Zhang et al., 2017). Nine arrays were present in *E. coli* isolates from beef carcasses, including *linF–aadA2*, *dfrA17–aadA5*, *aadB–blaOXA-10*, *dfr12–orfF–aadA2*, *dfrA1–aadA1*, *dfrA12–aadA2*, *aadA2*, *dfr12*, and *aadB–aadA2* (Chen et al., 2017). Five arrays were in *E. coli* isolates from waterfowls (*dfrA1–orfC*, *aadA2*, *aadA1*, *dfrA1–aadA1*, and *dfrA1–orfC–aadA1*; Zhang et al., 2019b). The different arrangements of gene cassettes of *E. coli* isolates from healthy swine were not only reported in China but also in other countries. For instance, nine different gene cassette arrays were described in 393 intestinal *E. coli* isolates in Spain, with *dfrA1–aadA1* in a dominant position (Marchant et al., 2013). Ten gene cassette arrays were observed in 103 class 1 integron-positive *E. coli* from two commercial production facilities in New South Wales, Australia (Reid et al., 2017). These results highlighted the role of pork, beef, and poultry products as a potential source for MDR *E. coli* strains and the necessity for controlling animal product safety.

In this study, the common gene cassette arrays were still prevalent in MDR *E. coli*. High frequency of 11 simple gene arrays were found, including *dfrA17–aadA5*, *dfrA12–orfF–aadA2, dfrA1–aadA1*, *dfrA12–orfF*, *dfrA1–aadA2*, *aadA1–dfrA1–aadA2*, *aadA1–dfrA1*, *aadA1–aadA2*, and *aadA2*. Then, the complex gene cassette arrays exhibited more MDR patterns through multiple cassettes, hybrid cassettes, and recombination cassettes. Isolates could contain two, three, four, and five cassettes in a single array. The complexity of the cassette array is strongly correlated with MDR patterns and phenotype.

In addition, six isolates were identified carrying hybrid gene cassettes (gene cassettes arrays marked in lowercase letter b, in Table 3). Our results were in agreement with the reported array *dfrA12–orfF–aadA2* cassette, in which *aadA2* often was replaced by the corresponding part of the *aadA1* cassette (Alicia et al., 2005). Moreover, *aadA16–aadA5–dfrA17* was a novel array. To the best of our knowledge, this was the first time to report a relative higher incidence of complex cassettes prevailing in intensive-farming swine in China.

### Future Work Directions Complex Structure Arrays

We noticed that 12 isolates had a larger variable region containing four or five different gene cassettes, *dfrA12–orfF–aadA2–adA1–cmlA1* (*n* = 8), *dfrA12–orfF-aadA2/aadA2–aadA1–cmlA1* (*n* = 2), *dfrA12–orfF–aadA2/aadA1–cmlA1* (*n* = 1), and *dfrA12–orfF–aadA2/1* (*n* = 1). They were highly consistent with nonclassic integrons that Partridge (Partridge et al., 2009) summarized as the 5'-CS but not the typical 3'-CS or incomplete transposition (tni) region. Similar arrays have been reported in one *E. coli* isolate from retail meat (pork; Jiang et al., 2017b), two commensal *E. coli* strains from feces (Moran et al., 2016; Oliva et al., 2018), and three clinical *E. coli* strains from different sources (samples from patients, dogs, swine, food products, and environment; Antunes et al., 2007; Sa’enz et al., 2010; María et al., 2011; Siqueira et al., 2016). In addition, four gene arrays did not exhibit their corresponding MDR phenotypes. This may due to the fact that many would affect the expression of gene cassettes, such as the strict regulation of integrase expression, the strength of the Pc promoter, the gene cassette arrangement, and the antibiotic concentrations (Vinué et al., 2011). Therefore, our further work will focus on these special arrays and their roles in conferring antibiotic resistance.

In conclusion, this study reported that the prevalence of antimicrobial resistance and the presence of class 1 integrons of commensal *E. coli* in the present four provinces were lower than previously reported in other regions. A high number of commensal *E. coli* with class 1 integrons were widely distributed in investigated farms, indicating a higher incidence of lateral resistant gene-transfer events. What is more, a portion of commensal *E. coli* harboring diverse cassette arrays contributed to their MDR phenotypes. Our finding indicates that the nonclinical swine raised in intensive farm would be a reservoir of MDR *E. coli*, which is a potential health risk.

## Data Availability Statement

The raw data supporting the conclusions of this article will be made available by the authors, without undue reservation.

## Author Contributions

KG and YZ conceived and designed the study. XZ performed the data analyses and wrote the manuscript. XL and JQ performed antimicrobial susceptibility testing and molecular experiments. WW, DW, and YL collected the samples and isolated *Escherichia coli*. LX reviewed and edited the manuscript. All authors contributed to the article and approved the submitted version.

### Conflict of Interest

The authors declare that the research was conducted in the absence of any commercial or financial relationships that could be construed as a potential conflict of interest.

## Footnotes

^1^http://www.ncbi.nlm.nih.gov/BLAST

^2^http://integrall.bio.ua.pt
